# The *Salmonella* effector protein SifA plays a dual role in virulence

**DOI:** 10.1038/srep12979

**Published:** 2015-08-13

**Authors:** Weidong Zhao, Thomas Moest, Yaya Zhao, Aude-Agnès Guilhon, Christophe Buffat, Jean-Pierre Gorvel, Stéphane Méresse

**Affiliations:** 1 Centre d’Immunologie de Marseille-Luminy, Aix Marseille Université UM2, Inserm, U1104, CNRS UMR7280, 13288 Marseille, France.; 2 URMITE, Aix Marseille Université, CNRS UMR 6236-IRD 198, Marseille, France; 3 Laboratoire de Biochimie et de Biologie Moléculaire, Hôpital de la Conception, Marseille, France

## Abstract

The virulence of *Salmonella* relies on the expression of effector proteins that the bacterium injects inside infected cells. *Salmonella* enters eukaryotic cells and resides in a vacuolar compartment on which a number of effector proteins such as SifA are found. SifA plays an essential role in *Salmonella* virulence. It is made of two distinct domains. The N-terminal domain of SifA interacts with the host protein SKIP. This interaction regulates vacuolar membrane dynamics. The C-terminal has a fold similar to other bacterial effector domains having a guanine nucleotide exchange factor activity. Although SifA interacts with RhoA, it does not stimulate the dissociation of GDP and the activation of this GTPase. Hence it remains unknown whether the C-terminal domain contributes to the function of SifA in virulence. We used a model of SKIP knockout mice to show that this protein mediates the host susceptibility to salmonellosis and to establish that SifA also contributes to *Salmonella* virulence independently of its interaction with SKIP. We establish that the C-terminal domain of SifA mediates this SKIP-independent contribution. Moreover, we show that the two domains of SifA are functionally linked and participate to the same signalling cascade that supports *Salmonella* virulence.

The Gram-negative bacterium *Salmonella* Typhimurium is an intracellular pathogen whose virulence relies on the capacity to survive and replicate inside cells of the infected host. The intracellular phase requires the expression of the type 3 secretion system-2 (T3SS-2), which is expressed by the intracellular bacterium in response to the vacuolar environment^1^. T3SS-2 mediates the translocation across the vacuolar membrane of a set of bacterial effector proteins that support collectively the intra-vacuolar replication (for review see^2^).

The T3SS-2 effector protein SifA^3^ plays a significant role in *Salmonella* virulence and several cellular phenotypes are linked to its translocation. SifA is required to maintain the integrity of the *Salmonella*-containing vacuole (SCV)^4^. It promotes the formation of tubular membranous structures connected to SCVs that are named *Salmonella*-induced tubules^5^,^6^,^7^,^8^.

Several functional peptide stretches and domains of SifA have been identified. The N-terminal residues direct the T3SS-2-mediated secretion/translocation of this effector^9^. The last C-terminal residues form a CAAX motif, which is found in eukaryotic Rab GTPase but also in many bacterial effectors^10^. Following translocation, the CAAX motif of SifA is isoprenylated and S-acylated by the eukaryotic enzymatic machinery^11^ and these modifications enable the membrane anchoring of the effector^12^. The resolution of the crystal structure of SifA has shown that the protein is divided into two distinct major domains^13^,^14^ separated by a potential caspase-3 cleavage site^15^. Thus, the two domains of SifA might act independently of each other upon cleavage. However, the caspase-3-mediated cleavage of SifA has not been demonstrated.

The SifA N-term domain (residues 1 to 136) interacts with the eukaryotic proteins SKIP (Plekhm2)^16^,^17^, and Plekhm1^18^. SKIP itself interacts with kinesin-1^16^,^19^. This microtubule-based motor protein is recruited to the SVC and tubules by the *Salmonella* effector PipB2^20^. The interaction with SKIP is thought to relieve the auto-inhibition of kinesin-1 and to favour its engagement with microtubules^17^.

The SifA C-term domain has a fold similar to the *Salmonella* effector SopE, which is a guanine nucleotide exchange factor (GEF). SifA interacts preferentially with the GDP bound form of the RhoA GTPase^13^,^14^ but does not stimulate the nucleotide exchange for this GTPase^21^. Thus, the contribution of the C-term domain to the functions and the virulence mediated by this effector remains unknown.

Considering the critical role of SifA in *Salmonella* virulence and the fundamental importance of a complete knowledge of the signalling pathway (s) controlled by this protein, we decided to investigate further the role of the SifA C-term in the pathogenic process. For this, we took advantage of a *SKIP* knockout mouse model and of *Salmonella* strains expressing various point mutated or truncated version of SifA. The present study establishes that SKIP is an important mediator of SifA functions but also that the C-term domain of SifA plays a SKIP-independent function in virulence. The two domains perform distinct but complementary roles. Finally, we conclude that the two domains of SifA are functionally but not necessarily physically linked.

## Results

### Characterization of *SKIP* ^−/−^ mice

*SKIP*^−/−^ mice were obtained from the Sanger Institute that performs systematic phenotyping of knock-out mice^22^ and makes data available online^23^. *SKIP*^−/−^ mice are declared as presenting slight increases for leukocyte cell number and for level of circulating alkaline phosphatase in females and males, respectively. We analysed blood samples but did not find significant differences in the hematology profiles of 8-weeks-old females C57BL/6 versus *SKIP*^−/−^ mice (Supplementary Table S1). As an increased circulating alkaline phosphatase is a possible sign of liver dysfunction, we assayed two transaminases (AST and ALT) that are commonly use to monitor liver damage. Mean AST and ALT levels (18 unsexed mice in each group) were slightly higher in *SKIP*^−/−^ as compared to C57BL/6 mice (Supplementary Table S1), but these differences are not significant (*p* = 0.5 and 0.19, respectively).

**Figure 1:**
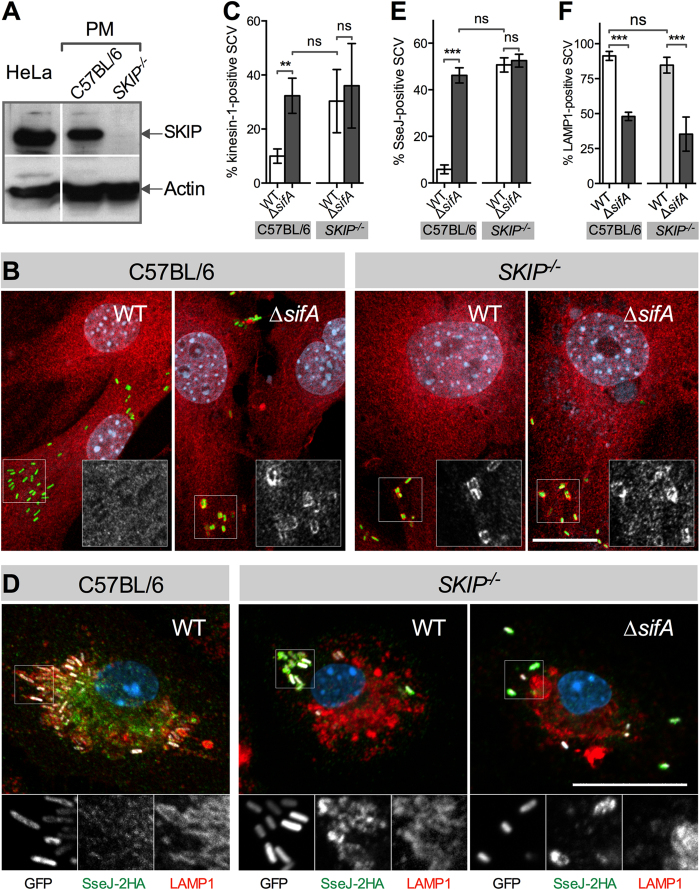
*Phenotyping of SKIP*^−/−^ *mice.* (**A**) *SKIP*^−/−^ mice do not express SKIP. Extracts from HeLa cells and peritoneal macrophages (PM) prepared from C57BL/6 or *SKIP*^−/−^ mice were examined for the presence of SKIP by Western blotting. Actin expression was used as a loading control. (**B**,**C**) SCVs accumulate kinesin-1 in the absence of SifA or SKIP. Mouse embryonic fibroblasts prepared from C57BL/6 or *SKIP*^−/−^ mice were infected with GFP-expressing wild type (WT) or ∆*sifA* strains of *Salmonella*. Cells were fixed 16 h post-infection and immunostained. (**B**) Cells were imaged by confocal microscopy for bacteria (green), kinesin-1 (red) and nuclei (light blue). Magnified insets showing single grayscale images for kinesin-1 are presented. (**C**) Percentages of kinesin-1-positive SCVs. Results are the means ± SD of three independent experiments. (**D**–**F**) SKIP is not essential for LAMP1 recruitment to the SCV. BMMs were prepared from C57BL/6 or *SKIP*^−/−^ mice and infected with wild type or ∆*sifA* strains of *Salmonella e*xpressing GFP from a plasmid and SseJ-2HA from the chromosome. Cells were fixed 16 h post-infection and immunostained. (**D**) BMMs were imaged by confocal microscopy for bacteria (white), SseJ (green), LAMP1 (red) and nuclei (light blue). Magnified insets showing single grayscale images for bacteria, SseJ and LAMP1 are presented. (**E** & **F**) Percentages of SseJ-positive (**E**) or LAMP1-positive (**F**) SCVs. Results are the means ± SD of three independent experiments. (**B** & **D**) Bar, 20 μm or 10 μm for the magnified insets. (**C**,**E** & **D**) Unpaired t-tests were used to compare two values. *P* values: ns, not significant; **P < 0.01; ***P < 0.001.

We used an anti-SKIP antibody^16^ to confirm the absence of the protein in *SKIP*^−/−^ mice. By Western blotting, the antibody recognizes in lysates of HeLa cells and of peritoneal macrophages derived from C57BL/6 mice a protein with an apparent molecular mass of ≈150 kDa that corresponds to SKIP. This protein was not detected in *SKIP*^−/−^ peritoneal macrophages (Fig. 1A).

The consequences of the lack of SKIP have been previously described in cultured cells using a siRNA-mediated knockdown^16^,^17^. In *Salmonella* infected HeLa cells, one observes an accumulation of kinesin-1 and of T3SS-2 effectors on SCVs in the absence of SifA or of SKIP. We checked these phenotypes using mouse embryonic fibroblasts and bone marrow-derived macrophages (BMM) prepared from C57BL/6 and *SKIP*^−/−^ mice and infected with wild type or ∆*sifA* strains of *S.* Typhimurium (*Salmonella enterica* subsp. *enterica*, strain NCTC 12023). A microscopic analysis of immunostained *SKIP*^−/−^ mouse embryonic fibroblasts showed that kinesin-1 accumulates both on wild type and ∆*sifA* SCVs (Fig. 1B). We found more than 30% of kinesin-1-positive SCVs in *SKIP*^−/−^ cells infected by one or the other strain (Fig. 1C). By contrast, in C57BL/6-derived cells, we detected 10 ± 2% of wild type SCVs decorated by the anti-kinesin-1 antibody as compared to 32 ± 6% for ∆*sifA* SCVs (Fig. 1C). Likewise, we observed in both cell types an accumulation of the T3SS-2 effectors SseJ and PipB2 (not shown) on SCVs in the absence of SifA or of SKIP (see Fig. 1D,E for SseJ in BMMs). We concluded that, as far as the accumulations of kinesin-1 and effectors are concerned, the consequences of the absence of SKIP are similar in *SKIP*^−/−^-derived cells and in HeLa cells.

### *SKIP* ^−/−^ mice are less sensitive to *Salmonella* infection than congenic C57BL/6

**Figure 2:**
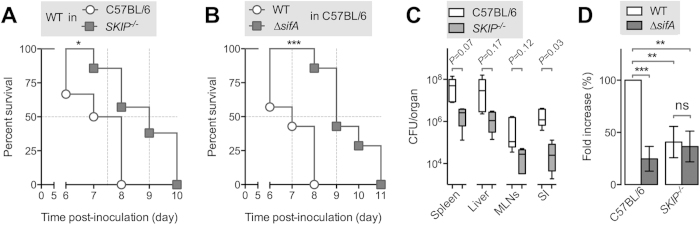
*SKIP*^−/−^ *mice are more resistant to salmonellosis than congenic C57BL/6 mice*. (**A**–**C**) Mice were inoculated P.O. with 10^5^ CFU of *S*. Typhimurium. (**A**) Survival of C57BL/6 or *SKIP*^−/−^ mice challenged with a wild type strain. (**B**) Survival of C57BL/6 challenged with a wild type or ∆*sifA* strain. (**C**) Bacterial loads in spleen, liver, mesenteric lymph nodes (MNLs) and small intestine (SI) of C57BL/6 (light box) and *SKIP*^−/−^ (dark box) mice 5 days post-inoculation (5 mice per group). The data are shown as a box and whisker plot. Boxes range from the 25th to the 75th percentile and are intersected by the median line. The whiskers embrace the lowest and highest values. Unpaired t tests were used to compare group means. Two-tailed *P* values are indicated. (**D**) Bone marrow macrophages prepared from C57BL/6 or *SKIP*^−/−^ mice were infected with a wild type or ∆*sifA* mutant strain and lysed at 2 or 16 h post-infection for enumeration of intracellular bacteria. The values shown represent the fold increase calculated as a ratio of the intracellular bacteria between 16 and 2 h and normalized to that of the wild type strain in C57BL/6-derived macrophages. Values are means ± SD of three independent experiments. Survival curves were compared using the logrank test and two-tailed *P* values are reported. Unpaired t-tests were used to compare two values. *P* values: *P < 0.05; **P < 0.01; ***P < 0.001.

To investigate whether SKIP mediates susceptibility to salmonellosis, we inoculated perorally (P.O.) C57BL/6 or *SKIP*^−/−^ mice with 10^5^ CFU of *S*. Typhimurium and monitored their survival. *SKIP*^−/−^ mice succumbed to wild type *Salmonella* infection significantly later than C57BL/6 mice. C57BL/6 and *SKIP*^−/−^ mice had a median survival time of 7.5 and 9 days, respectively (Fig. 2A). We obtained very similar results for wild type and ∆*sifA Salmonella* strains in C57BL/6 mice (Fig. 2B), with a median survival time of 7 and 9 days, respectively. The absence of either SifA or SKIP results in a longer survival of mice exposed to a *Salmonella* challenge.

We examined how the lack of SKIP could decrease the susceptibility of mice to a *Salmonella* challenge. At day five post-inoculation, we found lower bacterial counts in organs of *SKIP*^−/−^ as compared to C57BL/6 mice (Fig. 2C). The ratios of bacterial burdens between *SKIP*^−/−^ and C57BL/6 mice were 1:23, 1:32, 1:25 and 1:51 in the spleen, the liver, the mesenteric lymph nodes and the small intestine, respectively. Although statistical analysis revealed significance only for bacterial numbers in the small intestine (see *P* values in Fig. 2C), it suggests that the absence of SKIP limits *Salmonella* replication. We supported this point using BMMs prepared from the two mouse lineages. Cells were infected with wild type or ∆*sifA* strains and the fold increase of intracellular bacteria between 2 and 16 h after infection was determined. As expected, in C57BL/6 macrophages we observed a dramatic replication defect for the ∆*sifA* mutant with respect to the wild type strain (Fig. 2D). In contrast, we detected in *SKIP*^−/−^ macrophages a lower *Salmonella* replication and a no significant difference between the two bacterial strains. We concluded that SKIP is an important mediator of the role played by SifA in *Salmonella* intracellular replication. All together our data indicate that SKIP, by interacting with SifA, mediates susceptibility to salmonellosis.

### Characterization of a SKIP-independent function of SifA

**Figure 3:**
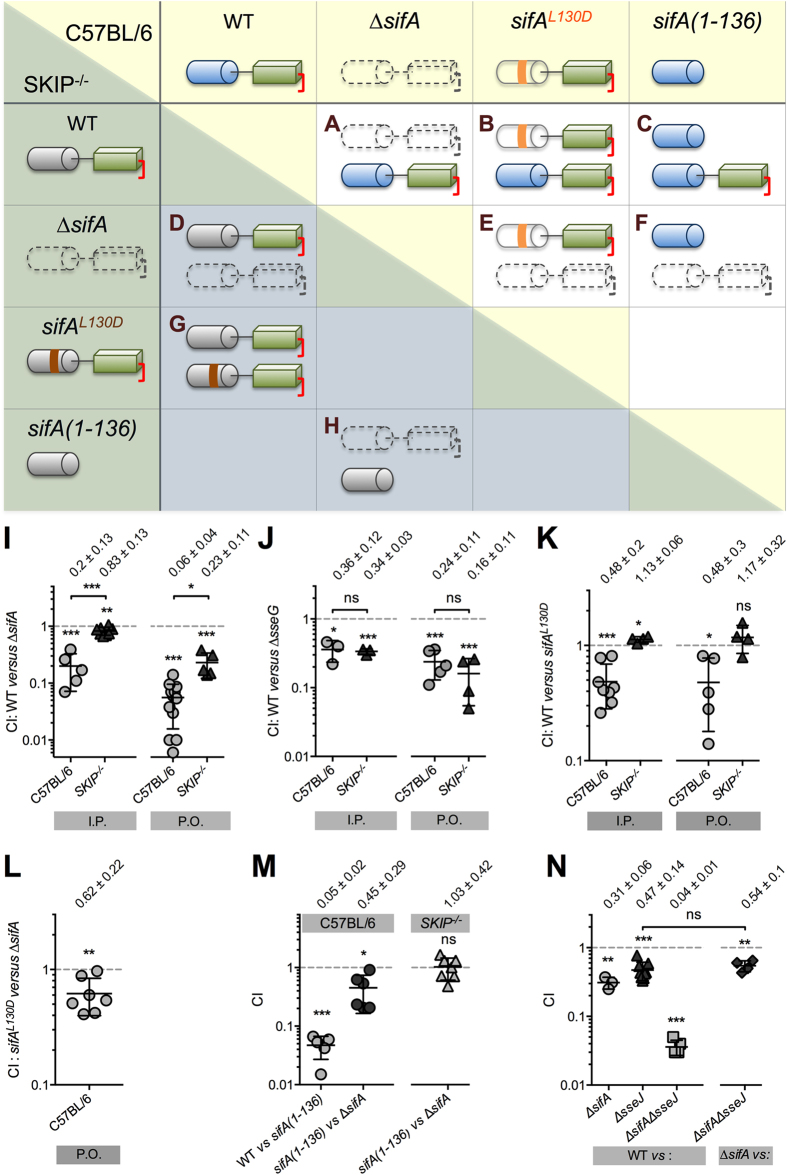
The SifA C-term is accountable for the SKIP-independent function of SifA in virulence. (**A**–**H**) Schematic representation of the SifA status in mixed infections of C57BL/6 (light, upper right part) or SKIP^−/−^ (shadowed lower left part) mice. The two-domain protein SifA is symbolized by a blue cylinder (N-term domain) connected to a green parallelepiped (C-term). The C-terminal CAAX motif is lipidated (red square bracket). A ∆*sifA* strain does not express the SifA protein (colourless/dotted line). SifA^L130D^ (colourless N-term) carries a mutation (L130D, in orange) that prevents the binding to SKIP. The strain *sifA(1-136)* expresses only the SifA N-term domain. As far as the interaction with SKIP is concerned this domain is not functional in *SKIP*^−/−^ mice (in grey). The different mixed infections were used to analyse the : (**A**) contribution of SifA; (**B** & **G**) SKIP-dependent contribution of SifA; (**C**) contribution of the SifA C-term; (**D** & **E**) SKIP-independent contribution of SifA; (**F**) contribution of the SifA N-term; (**H**) SKIP-independent contribution of the SifA N-term - to *Salmonella* virulence. (**I**–**N**) C57BL/6 or *SKIP*^−/−^ mice were inoculated I.P. or P.O. with 10^5^ CFU of various combinations of two *Salmonella* strains as indicated. CIs were determined two (I.P.) or five (P.O.) days post-inoculation. Each symbol represents one mouse. Mean CI ± SD values are represented and indicated on the top of each mouse group. (**I**) A ∆*sifA* strain is significantly attenuated in *SKIP*^−/−^ mice. (**J**) A ∆*sseG* strain presents the same attenuation of virulence in C57BL/6 and *SKIP*^−/−^ mice. (**K** & **L**) SifA plays a SKIP-independent function in virulence. (**M**) The C-term domain of SifA supports its SKIP-independent function in virulence. (**N**) *sifA* and *sseJ* contribute independently to *Salmonella* virulence. A one-sample t-test was used to determine whether a CI was significantly different of one, and unpaired t-tests to compare two values. *P* values: ns, not significant; *P < 0.05; **P < 0.01; ***P < 0.001.

Next, we examined if the role of SifA in virulence was exclusively mediated by its interaction with SKIP. For this purpose, we compared the virulence of diverse *Salmonella* strains in C57BL/6 and *SKIP*^−/−^ mice. Groups of mice were inoculated intraperitoneally (I.P.) or P.O. with different two strains combinations (1:1 mix) (Fig. 3) and bacteria were recovered from mouse spleens after two (I.P.) or five (P.O.) days to determine the competitive index (CI)^24^. We found that a ∆*sifA* mutant was still significantly attenuated as compared to wild type *Salmonella* in *SKIP*^−/−^ mice inoculated I.P. or P.O. (CI of 0.83 ± 0.13 and 0.23 ± 0.11, respectively) (Fig. 3A,D,I). As a control, we tested the virulence attenuation of a ∆*sseG* mutant^25^, which did not differ significantly between mice expressing or not SKIP (Fig. 3J). These data indicate that SifA mediates a SKIP-independent function in virulence.

**Figure 4:**
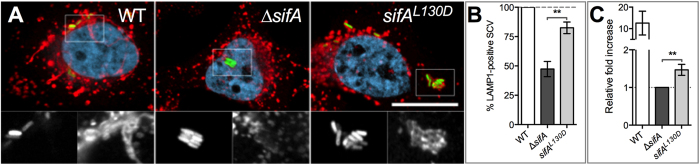
*SifA C-term function in infected cells*. HeLa cells (**A**,**B**) and RAW 264.7 mouse macrophages (**C**) were infected with wild type (WT) *S.* Typhimurium, a ∆*sifA* mutant or a *sifA*^*L130D*^ strain. (**A**,**B**) The SifA C-term increases the presence of LAMP1 on the SCV. (**A**) HeLa cells were infected for 14 h, immunostained and imaged for bacteria (green), LAMP1 (red) and DNA (light blue) using a confocal microscope. Magnified insets showing single labelling for GFP (left) and LAMP1 (right) are presented below each image. Bar, 20 μm or 10 μm for the magnified insets. (**B**) SCVs were scored for the presence or absence of LAMP1. Percentages were normalized to that of the wild type strain. Results are the means ± SD of three independent experiments. (**C**) The SifA C-term supports *Salmonella* intracellular replication. RAW 264.7 mouse macrophages were infected with different *Salmonella* strains and lysed at 2 or 16 h post-infection for enumeration of intracellular bacteria. The values shown represent the fold increase calculated as a ratio of the intracellular bacteria between 16 and 2 h and normalized to that of the ∆*sifA* strain. Values are means ± SD of four independent experiments. Unpaired t-tests were used to compare two values. *P* values: **P < 0.01.

SKIP binds the distal part of the SifA N-term. This interaction, which involves a network of hydrogen bonds and van der Waals contacts is abolished by the substitution of the interacting leucine 130 with an aspartic acid (L130D)^13^,^14^. This point mutated form of SifA is secreted and translocated by the T3SS-2^14^. To confirm that SifA acts also independently of its interaction with SKIP, we performed a mixed inoculation of wild type *Salmonella* and a strain expressing chromosomally SifA^L130D^ (Fig. 3B,G). The CIs in C57BL/6 mice inoculated I.P. or P.O. were both of  ≈0.5 (Fig. 3K). These results reveal the contribution of SKIP to the virulence mediated by SifA and indicate that this point mutant form of SifA is active despite its lack of interaction with SKIP and its propensity to be more rapidly degraded than the wild type protein in an eukaryotic environment (half-lives of 8 h and 5.3 h for SifA and SifA^L130D^, respectively, see Supplementary Fig. S1). In *SKIP*^−/−^ mice we obtained CI values of ≈1 (Fig. 3K), which ascertain that the CI values obtained in C57BL/6 mice are strictly reflecting the contribution of SKIP to the virulence mediated by SifA. We also infected C57BL/6 mice with a mix of *sifA*^*L130D*^ and ∆*sifA* strains (Fig. 3E) and observed a CI of 0.62 ± 0.22 (Fig. 4L) that substantiates a SKIP-independent function in virulence for SifA.

### The SKIP-independent function of SifA is associated with its C-term domain

To determine which of the N- or C-term domain of SifA is responsible for the SKIP-independent function in virulence, we constructed a *Salmonella* strain expressing chromosomally SifA deleted of its C-term domain [*sifA(1-136)*]. As small deletions throughout SifA are sufficient to block its secretion by T3SS-2^26^, we first verified that SifA(1-136) was secreted. For this, we engineered strains expressing SifA with an internal double haemagglutinin tag at the boundary between the N- and C-term domains and deleted [*sifA(1-136)-2HA*] or not (*sifA-2HA*) of its C-term domain. SifA(1-136) was efficiently secreted by the T3SS-2 (Supplementary Fig. S2) and as stable as SifA (Supplementary Fig. S1). These strains were tested in mixed P.O. infections. We found that as compared to wild type *Salmonella*, the *sifA(1-136)* mutant presented in C57BL/6 mice a strong attenuation of virulence similar to that of a ∆*sifA* strain (CI = 0.05 ± 0.02, Fig. 3C,M). Therefore, we compared the virulence of *sifA(1-136)* and ∆*sifA* mutant strains (Fig. 3F,M) and obtained in C57BL/6 mice a CI of 0.45 ± 0.29. This value is similar to that found for the SKIP-dependent role of SifA to virulence (Fig. 3B,K), suggesting that the contribution to virulence of the SifA N-term is solely mediated by SKIP. In *SKIP*^−/−^ mice (Fig. 3H) this mixed infection gave a CI of ≈1 (1.03 ± 0.42, Fig. 3M) that validated the hypothesis. We concluded that the SKIP-independent function observed in other mixed infections (Fig. 3D,E) is borne by the SifA C-term domain.

### SifA and SseJ contribute independently to *Salmonella* Virulence

The T3SS-2 effector SseJ exerts a lipase activity that increases the esterification of cholesterol in host cell membranes^27^. SseJ is activated by binding the GTP-bound form of the eukaryotic RhoA GTPase^28^ while SifA binds preferentially GDP-bound RhoA. Though a GEF activity of SifA toward RhoA could not been demonstrated^21^, it has been proposed that SifA participates to the activation of SseJ by favouring the recruitment of RhoA to the SCV and possibly its activation^29^. Thus, we tested whether the role of the SifA C-term in virulence could be linked to this proposed activity. In I.P inoculated mice we compared the virulence attenuation of strains deleted of *sseJ*, *sifA* or both genes. We found that a ∆*sifA*∆*sseJ* strain is more attenuated than each individual mutant as compared to wild type *Salmonella* (Fig. 3N) and that the CIs of ∆*sifA* versus ∆*sifA*∆*sseJ* is not different from the CI of wild type versus ∆*sseJ* (0.54 ± 0.01 and 0.47 ± 0.16, respectively). These findings suggest that the two genes are involved in distinct signalling pathways. Therefore the function of the SifA C-term in the mouse model of infection is probably not linked to function of RhoA-SseJ in virulence.

### The SifA C-term is important for the recruitment of LAMP1 to the SCV and for intracellular replication

We finally investigated how the C-term domain contributes to the SifA function. Compared to the wild type, the ∆*sifA* SCV is characterized by very low levels of lysosomal glycoproteins^4^. Thus, we explored whether the SifA C-term could be important for the recruitment of these membrane proteins to the SCV. Firstly, we explored this phenotype in BMMs prepared from C57BL/6 and *SKIP*^−/−^ mice and infected with wild type or ∆*sifA Salmonella* strains. The presence of LAMP1 on SCVs at 16 hours post-infection was examined and illustrated by confocal microscopy (Fig. 1D,F). Most wild type SCVs were LAMP1-positive in macrophages expressing or not SKIP (91 ± 3% and 85 ± 6%, respectively) while the fraction of ∆*sifA* SCVs decorated by the anti-LAMP1 was below 50% in both types of macrophages (48 ± 3% *versus* 35 ± 12%). This indicates that the recruitment of LAMP1 requires SifA but not its interaction with SKIP. To confirm this result, we infected HeLa cells with the same *Salmonella* strains and also a strain expressing chromosomally SifA^L130D^. We observed that expression of SifA^L130D^ increased noticeably the fraction of LAMP1-positive SCVs (Fig. 4A,B) thus confirming the prominent role of the SifA C-term in this phenotype.

This result prompted us to compare the capacity of these various strains to replicate intracellularly. These experiments were performed in mouse macrophages as strains deleted of *sifA*^4^ or expressing SifA^L130D^^14^ partly escape the vacuole and replicate well in the epithelial cell cytosol^30^. We found that the expression of SifA^L130D^ increases the intracellular bacterial growth by a factor ~1.5 (Fig. 4C). Although modest this increase was significant. Collectively, these results establish that the SifA C-term supports the recruitment of LGPs to SCVs. This is associated with a fairly small but robust positive effect on *Salmonella* intracellular replication.

## Discussion

For this study, we developed a *SKIP*^−/−^ mouse model of acute *Salmonella* infection. It helped us to answer the question of whether the virulence mediated by SifA is only supported by its interaction with SKIP and subsequently whether the C-terminal domain of SifA is functional on its own. Our results show that both the SifA N-term *via* SKIP and the SifA C-term play a role during *Salmonella* infection. Remarkably, the two domains are probably involved in the same signalling cascade.

*SKIP*^−/−^ mice were maintained as a colony of homozygote animals. These mice are viable, have a good prolificity and do not present signs of disease or debility. Thus, SKIP is dispensable for the life of C57BL/6 mice indicating that in laboratory stabling conditions the function of this protein is not required and/or that another protein is capable of complementing the functions of SKIP. At the cellular level we did not observed the Golgi scattering^12^ or the clustering of the LAMP1 compartment^31^ that were previously described upon siRNA-mediated knock-down of SKIP expression. Thus, the function of SKIP regarding the positioning and the organization of these organelles is complemented and these results rather support the hypothesis of a protein compensating for the lack of SKIP.

We found that *SKIP*^−/−^ mice are more resistant to a *Salmonella* challenge than congenic C57BL/6 mice and we observed very similar profiles for the survival curves of wild type *Salmonella* in *SKIP*^−/−^ mice and for a ∆*sifA* strain in C57BL/6. This SKIP-mediated susceptibility to salmonellosis likely reflects the role played by the SifA-SKIP interaction during the infection. The SifA-SKIP complex interacts and activates kinesin-1 and thereby favours the formation of vesicles and tubules^17^. By sequestering rab9, it also inhibits the retrograde transport of mannose 6-phosphate receptors to the *trans*-Golgi network and the delivery of lysosomal enzymes to lysosomes^32^. Both functions are probably affected by the absence of SKIP and their deficiencies contribute to the decreased susceptibility of *SKIP*^−/−^ mice to salmonellosis.

These results are seemingly contradictory with the higher susceptibility of *SKIP*^−/−^ mice to a *Salmonella* challenge reported online by the Sanger Institute. This laboratory infects mice I.V. with a sub-lethal dose of an attenuated strain of *S.* Typhimurium. In these conditions, infected C57BL/6 mice survive and resolve the infection within approximately one month while they succumb within one week when challenged P.O. or I.P. with the dose of *S*. Typhimurium 12023 used in this study (Fig. 2A). A rough estimation based on the Sanger Institute’s data indicates that bacterial loads in the liver and the spleen fourteen days post I.P. inoculation are several logs (>4) lower than those we observed after two days. Therefore, SKIP is required for acute infection of susceptible mice by a virulent *Salmonella* strain while it is necessary for the host resistance during later stage of infection by an attenuated *Salmonella* strain.

By performing *in vivo* competitions of *Salmonella* strains we found that SifA exerts a SKIP-independent function that is borne by its C-term domain. Hence, SifA has two domains and two functions. A comprehensive analysis of the CI results emphasizes other important information regarding the functional and physical interactions between the two domains.

A putative caspase 3 cleavage site separates the N- and C-term domains of SifA^15^ but it is not known whether the two domains are really split apart after SifA translocation. We assessed the role of the SifA N-term domain in virulence in the context of a membrane bound (Fig. 3B,K, WT *versus sifA*^*L130D*^) or cytosolic (Fig. 3F,M, *sifA[1-136] versus* ∆*sifA*) protein. In both cases we obtained CI values of ≈0.5 indicating that the N-term domain of SifA is equally functional in either context. Therefore, these data do not exclude the possibility that translocated SifA is cleaved.

The consequences of the absence of one or the other domain of SifA on virulence are markedly different. While the lack of a functional N-term domain impacts moderately the virulence of *Salmonella* (Fig. 3B,K, CI ≈ 0.5), the absence of the C-term domain (Fig. 3C,M, CI ≈ 0.05) diminishes dramatically the virulence of the strain. Thus, despite its lack of characterization, the C-term is clearly crucial for SifA functions. The role in virulence of this domain was analysed in the context of a fully functional molecule (Fig. 3C,M, WT versus *sifA[1-136]*) or in the absence of a functional N-term domain (Fig. 3E,L, *sifA*^*L130D*^*versus* ∆*sifA,* and Fig. 3D,I, WT *versus ∆sifA* in *SKIP*^−/−^ mice). The CI values were considerably divergent and this observation reveals that the contribution of the SifA C-term to virulence is far more important in the presence of a functional N-term domain. The SifA C-term is poorly functional in the absence of SKIP or a N-term domain capable of interacting with SKIP and this entails that the SifA C-term acts upstream of the SifA-SKIP signalling cascade. Therefore, it appears that the two domains of SifA are functionally but not necessarily physically linked.

Very recent studies have highlighted the role of the host protein Plekhm1 in *Salmonella* infection. This protein interacts with the SifA N-term domain and supports *Salmonella* intracellular replication^18^. These results are apparently inconsistent with our present data indicating that SKIP only mediates the functions of the SifA N-term domain. One can hypothesise that Plekhm1 is unnecessary for the virulence in the mouse model of infection or that Plekhm1 needs SKIP to play its role in the infectious process. This last hypothesis is however unlikely as the two proteins are competing for binding SifA^18^.

We have previously shown that the plasmidic expression *sifA*^*L130D*^ in a ∆*sifA* strain increases the frequency of mouse macrophages enclosing more than 10 bacteria^14^ and this suggested that SifA^L130D^ supports *Salmonella* replication. The present study confirms this observation. In addition, we observed that SifA^L130D^ increases the proportion of LAMP1-positive SCVs. Owing to the increased recruitment of LGPs, the *sifA*^*L130D*^ SCV is probably more stable and it might explain the slightly better replication of this *Salmonella* strain in macrophages. SifA^L130D^ is more rapidly degraded than wild type SifA in a eukaryotic context, may be because of its lack of interactions with SKIP. Thus, our results may underestimate the functional impact of the SifA C-term. This possibility is however balanced by the consistency of our observations regarding the composition of the SCV and the virulence for the *sifA*^*L130D*^ mutant and the wild type strain in the absence of SKIP.

Considering the GEF-like conformation of the SifA C-term^13^,^14^, we questioned the function of this domain and paid a special attention to its possible interaction with host GTPases. SifA binds the GDP-bound RhoA and may support the SseJ function by recruiting the GTPase to the SCV membrane^29^. Our results do not support this hypothesis as we found that the products of *sifA* and *sseJ* contribute independently to *Salmonella* virulence in the mouse model of infection. However, the *sifA* and *sseJ* interaction might be host-specific.

The present study reveals that the two domains of SifA are functional and participate to the same pathway supporting the important role of SifA in virulence. Considering the GEF-like structure of the SifA C-term, this domain likely interacts and modulates the activity of a GTPase. Studies to identify this protein may provide important insights into the mechanisms by which SifA regulates the membrane dynamics of the bacterial vacuole.

## Methods

### Material

A hematology analyser (HORIBA ABX Pentra 60 C, calibrated for mouse cells) was used for analysis of whole-blood specimens. Plasma AST and ALT activities were assayed using a Cobas C 501/502 analyser (Roche Diagnostics). For quantification of western blotting, chemiluminescence signal was read with an Azure biosystems C300.

### Antibodies

HA-tagged proteins were detected using a mouse monoclonal anti HA (Covance, clone 16B12). A rabbit polyclonal serum was used to detect SKIP^16^. The rabbit anti-kinesin HC (PCP42) (a kind gift from R. Vale) was absorbed with *Salmonella* acetone powder^33^. Secondary antibodies for Western blotting were goat anti-mouse or anti-rabbit IgG HRP conjugate (Sigma–Aldrich). Fluorescent Alexa secondary antibodies were obtained from Jackson ImmunoResearch

### Mouse Strains

C57BL/6 were obtained from Charles River Laboratory. *SKIP*^−/−^ mice (B6N;B6J-Tyr^c-Brd^ Plekhm2^tm1a(EUCOMM)Wtsi/Wtsi^) were obtained from the Wellcome Trust Sanger Institute (EUCOMM Consortium). A mouse colony was maintained by incrossing homozygotes, which had been genotyped as described by the Sanger Institute.

### Ethic statement

Animal experimentation was conducted in strict accordance with good animal practice as defined by the French animal welfare bodies (Law 87–848 dated 19 October 1987 modified by Decree 2001-464 and Decree 2001-131 relative to European Convention, EEC Directive 86/609). All animal work was approved by the Direction Départementale des Services Vétérinaires des Bouches du Rhône (authorization number 13.118 to SM, Application number AR 1A09382857717).

### Bacterial strains and growth conditions

Bacterial strains used in this study are listed in SI Table 1. Strains were cultured in LB broth (Difco) or minimal medium (M9, glycerol 0.2%, MgSO_4_ 1 mM, CaCl_2_ 200 mM, thiamine 1 mg/ml, casamino acids 1 mg/ml). The T3SS-2 effetor secretion assays were conducted as described by Yu *et al.*^34^. Briefly, bacteria were sub-cultured in MgM-MES pH 5, for 4 hours. Secretion was triggered by a pH shift (MgM-MES pH 7.2). Ampicillin (50 μg/ml), kanamycin (50 μg/ml), tetracycline (10 μg/ml) and chloramphenicol (50 μg/ml) were added when required.

### Construction of plasmids

Point mutation of plasmids were carried out by PCR amplification of the entire plasmid DNA with mutagenic primers divergently oriented and overlapping at their 5′ ends^35^. The oligo pairs O-461/O-466 and O-464/O-466 (Supplementary Table S2) were used to introduce the L130D mutation in SifA or SifA-2HA, respectively.

### Construction of mutant strains

Non-polar gene-deletion mutants were generated by the lambda Red recombinase system^36^, using gene-specific primer pairs to amplify the pKD4 kanamycin resistance gene as described in Supplementary Table S2. Mutagenesis was performed in a 12023 strain background. When necessary *Salmonella* mutants were transformed with the pCP20 plasmid to excise the antibiotic cassette. A strain expressing SifA-2HA from the chromosome (WZ012) was produced as described elsewhere^37^. The amplicons of pKD4 with the oligo pairs O-601/O-602 or O-744/O-71 were recombined in the wild type strain to obtain the ∆*ssaV*::Km^R^ (AA057) or *sifA(1-136)*::Km^R^ (WZ020) mutant strain, respectively. The amplicon of pKD4 with the oligo pairs O-745/O-71 was recombined in the *sifA-2HA*sc4 strain (WZ012sc4) to obtain the *sifA(1-136)-2HA*::Km^R^ (WZ019). For WZ039, the amplicon of pKD4-SifA^L130D^-2HA with oligos O-686 and O-687 was used to perform a chromosomal recombination in a ∆*sifA*::FRT strain. For WZ041 (*sifA*-2HA, ∆*ssaV*), a P22 lysate of the strain AAG057 was used to transduce ∆*ssaV*::Km^R^ into the WZ012sc4 strain. WZ042 and WZ043 strains were obtained by P22-mediated transduction of ∆*sifA*::Km^R^ or *pipB2-2HA*::Km^R^ into a ∆*sseJ* strain, respectively.

### Eukaryotic cells and culture conditions

RAW 264.7, HeLa, primary bone marrow-derived macrophages and embryonic fibroblasts were grown in DMEM (GibcoBRL) supplemented with 10% foetal calf serum (FCS; GibcoBRL), 2 mM nonessential amino acids, and glutamine (GibcoBRL) at 37 °C in 5% CO_2_. For peritoneal macrophages, mice (C57BL/6 or *SKIP*^−/−^) were injected intraperitoneally with a thioglycollate solution for a volume of 1 ml per mouse. Four days post injection, the thioglycollate-pretreated mice were sacrificed and macrophages were harvested form the peritoneal cavity by washing with 5 ml of cold PBS. Cells were collected from the washing solution by centrifugation, washed again twice with cold PBS, resuspended in DMEM based growing medium and seeded at a density of 10^5^ cells per cm^2^ in 6- or 24-well plates. Cells were used for infection after 1 day culture. For preparing mouse embryonic fibroblasts C57BL/6 or *SKIP*^−/−^ mice were sacrificed at 13–14 days gestation. The uterine horns were collected and washed 3 times with 10 ml PBS. Then, visceral tissues were separated from embryos. Embryos were washed again for 3 times with PBS and then finely minced with a curved dissecting scissors. A volume of 2 ml of trypsin was added and incubated for 5 min, during which the tissue was minced. 5 ml trypsin were added and the cells were pipetted vigorously up and down. The cells were placed into incubator for 20–30 min and again pipetted vigorously up and down. Cells were diluted in DMEM-derived growing medium and seeded in 75 cm^2^ flasks and incubated at 37 °C in a tissue culture incubator until the flasks are at least 90% confluent. Then, the cells were split using trypsin and seeded in 6- or 24-well plates for infection.

### Bacterial infection and replication assays

Bone marrow-derived macrophages, HeLa and RAW 264.7 macrophages were grown, infected and treated as previously described^7^.

### Competitive index

C57BL/6 or congenic *SKIP*^−/−^ mice (eight to ten weeks old) were inoculated intraperitoneally or perorally with equal amounts of two bacterial strains for a total of 10^5^ bacteria per mouse. The spleens were harvested two (I.P.) or five (P.O.) days after inoculation and homogenized. Bacteria were recovered and enumerated after plating a dilution series onto LB agar with the appropriate antibiotics. Competitive indexes (CI) were determined for each mouse^38^,^24^. The CI is defined as the ratio between the mutant and wild type strains within the output (bacteria recovered from the mouse after infection) divided by their ratios within the input (initial inoculum).

### Statistical Analyses

Statistical analyses were performed with Prism 5 software (GraphPad) with one-way ANOVA and Tukey post-test or two-tailed unpaired Student’s t test. *P*-values: ns, not significant; *P < 0.05; **P *<* 0.01; ***P < 0.0005.

## Additional Information

**How to cite this article**: Zhao, W. *et al.* The *Salmonella* effector protein SifA plays a dual role in virulence. *Sci. Rep.* **5**, 12979; doi: 10.1038/srep12979 (2015).

## Supplementary Material

Supplementary Information
