# Supplementary material for: The Salmonella effector protein SifA plays a dual role in virulence
Source: Sci Rep. 2015 Aug 13;5:12979. doi: 10.1038/srep12979 (PMC4534788; doi:10.1038/srep12979)
Supplement: Supplementary Information [file srep12979-s1.pdf]

Supplementary information

The *Salmonella* effector protein SifA plays a dual role in virulence

Weidong Zhao[1], Thomas Moest[1], Yaya Zhao[1], Aude-Agnès Guilhon[1], Christophe Buffat[2,3], Jean-Pierre Gorvel[1] and Stéphane Méresse[1,*]

[1]Centre d'Immunologie de Marseille-Luminy, Aix Marseille Université UM2, Inserm, U1104, CNRS UMR7280, 13288 Marseille, France.

[2]URMITE, Aix Marseille Université, CNRS UMR 6236-IRD 198, Marseille, France

[3]Laboratoire de Biochimie et de Biologie Moléculaire, Hôpital de la Conception, Marseille, France

*Corresponding author: Stéphane Méresse; meresse@ciml.univ-mrs.fr; Tel: 33(0)4 91 26 91 15; Fax: 33(0)4 91 26 94 30

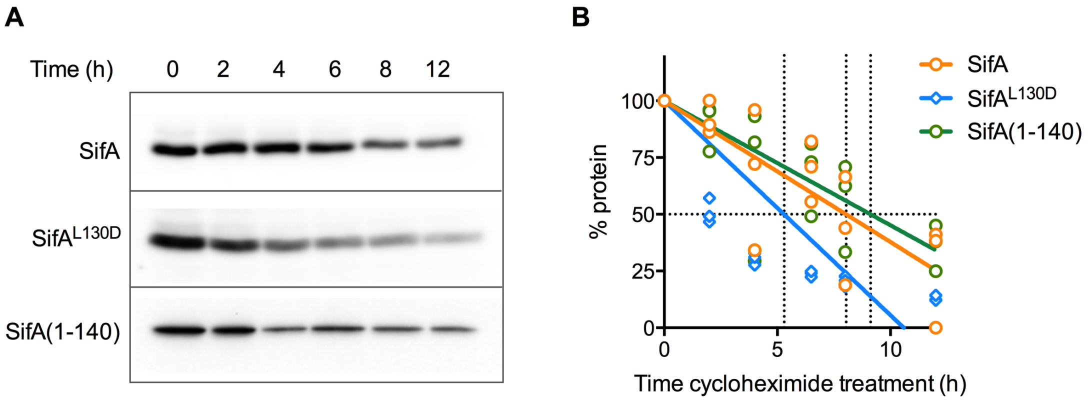

Fig. S1. *Stability of ectopically expressed mutant forms of SifA*. HeLa cells cells were transfected with vectors for expression of HA-tagged version of SifA, SifA^{L130D} or SifA(1-140) for 24 h. Cells were then treated with cycloheximide (100 µg/ml) for 0, 1, 2, 4, 8 and 12 hours to inhibit protein synthesis. Samples were examined for the presence of HA-tagged proteins by Western blotting. (**A**) Membranes were imaged using a chemiluminescence system. Representative image are shown. (**B**) The level of protein at each time point was quantified using the ImageJ software. The level of each protein at time 0 was set as 100% and the percentage of protein remaining at each time point was calculated and plotted. Three independent experiments are plotted. Linear regression analysis of each data set was carried out using the Prism software and used to calculate the proteins half-lives (8, 5.3, 9.1 h for SifA, SifA^{L130D} and SifA(1-140), respectively).

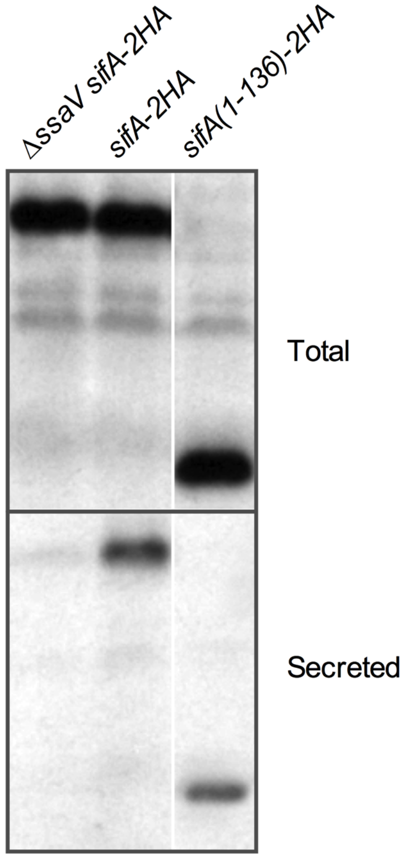

Fig. S2. *A C-terminally truncated form of SifA is secreted*. Strains expressing full length SifA-2HA or SifA(1-136)-2HA were tested *in vitro* for the secretion of effectors. A strain expressing SifA-2HA but unable to secrete T3SS-2 effectors (Δ*ssaV*, *sifA-2HA*) was used as negative control.

Table S1. *Blood parameters for C57BL/6 and SKIP$^{-/-}$ mice*. Hematological parameters were measured in groups (n=6) of 8 weeks C57BL/6 and *SKIP*$^{-/-}$ females using the hematology analyzer ABX Pentra 60 C (HORIBA ABX Diagnostic) calibrated for mouse cells. The plasma activities of aspartate (AST) and alanine aminotransferase (ALT) were determined in groups of 18 unsexed C57BL/6 and *SKIP*$^{-/-}$ mice using a fully automated clinical chemistry analyser (Cobas C 501/502 analyser, Roche Diagnostics, Germany) and commercially available reagent kits. Unpaired t-tests were used to compare group means. *P* values are indicated.

	C57BL/6	*SKIP*$^{-/-}$	*P* value
**Hematology**			
White blood cells (10^3/mm^3)	8,32 ± 1.34	8.05 ± 1.31	0.98
Lymphocytes (%)	70.08 ± 3	76.01 ± 4.3	0.70
Monocytes (%)	6.28 ± 1.7	5.33 ± 0.66	0.95
Neutrophils (%)	22.45 ± 1.92	17.16 ± 3.51	0.73
Eosinophils (%)	0.60 ±0.16	0.95 ± 0.48	0.98
Basophils (%)	0.58 ± 0.31	0.53 ± 0.18	0.99
Atypical lymphocyte (%)	0.56 ± 0.12	0.63 ± 0.23	0.99
Large immature cells (%)	0.70 ± 0.21	0.55 ± 0.24	0.99
Red blood cells (10^6 / mm^3)	9.4 ± 0.27	8.99 ± 0.31	0.97
Hemoglobinemia (g/d)	15.26 ±0.43	14.4 ± 0.56	0.95
Hematocrit (%)	48.55 ± 1.36	46.36 ± 1.57	0.88
mean corpuscular volume (µm^3)	51.66 ± 0.51	51.5 ± 1.04	0.99
Mean Corpuscular Hemoglobin (pg)	16.21 ± 0.09	15.98 ± 0.16	0.98
Mean Corpuscular Hemoglobin Concentration (g/d)	31.43 ± 0.2	31.06 ± 0.42	0.98
Red cell distribution width (%)	11.95 ± 0.61	12.08 ± 0.25	0.99
Platelet counts (10^3/mm^3)	686 ± 12	702 ± 78	0.03
Mean platelet volume (µm^3)	5.63 ± 0.07	5.73 ± 0.29	0.99
**Clinical chemistry**			
AST (U/l)	139 ± 104	170 ±157	0.5
ALT (IU/l)	39 ± 25	57 ± 52	0.19

Table S2. Salmonella strains and plasmids

Name	Description	Reference
	**Strains**	
12023	Wild-type *S*. Typhimurium (*Salmonella enterica* subsp. *enterica*, strain NCTC 12023)	38
DH215K	12023 Δ*sifA*::Km^R	20
DH215sc4	12023 Δ*sifA*::FRT	20
ΔsseG	12023 Δ*sseG*::aphT	25
WZ012	12023 *sifA-2HA*::Km^R	37
WZ012sc4	12023 *sifA-2HA*::FRT	This study
AAG057	12023 Δ*ssaV*::Km^R	This study
WZ039	12023 $sifA^{L130D}$-2HA ::Km^R	This study
WZ020	12023 sifA(1-136)::Km^R	This study
WZ019	12023 sifA(1-136)-2HA::Km^R	This study
WZ041	12023 *sifA-2HA* Δ*ssaV*::Km^R	This study
WZ043	ΔsseJ pipB2-2HA::Km^R	This study
WZ042	Δ*sseJ* Δ*sifA*::Km^R	This study
	**Plasmids**	
pKD4-SifA-2HA, C987	pKD4 derivative for chromosomal exchange of *sifA* by *sifA-2HA*	37
pKD4-$SifA^{L130D}$-2HA, C988	pKD4 derivative for chromosomal exchange of *sifA* by *$sifA^{L130D}$-2HA*	This study

Table S3. Oligonucleotides

Primer	Sequence	Intend
O-461	GCTGTGAAGTCATGGGGAATCTTTATTTTGATATGAAAGATCGCCCGG ATATTTTAAAATC	*sifA*^{L130D}
O-464	GCTGTGAAGTCATGGGGAATCTTTATTTTGATATGAAAGATCGCCCGG ATTATC	*sifA*^{L130D}
O-466	ATTCCCCATGACTTCACAGCTAAAGGTATCGGTATTTTG	*sifA*^{L130D}
O-601	CTGGAGTCGCAATGCGTTCATGGTTAGGTGAGGGAGTCAGGGCGCG TGTAGGCTGGAGCTGCTTC	ΔssaV
O-602	GGCTCTCCTCTCCTAATTCCTGCCATGACAAAATCGGTACGTCGCATA TGAATATCCTCCTTAG	ΔssaV
O-686	CTGATTGCCAGTCTCTTTTAAAAATTATATTACATCCGATGCGCCCGC AGTTGAGATAAAAAGGG	WZ039
O-687	GGCCATTTAAATGACTATTCTCATCCGATCCGGTCATATGCGGCCATA TGAATATCCTCCTTAG	WZ039
O-71	GCCTGGCAAGAGGTTACTCAGTAGGCAAACAGGAAGCATATGAATAT CCTCCTTAG	WZ020, WZ019, WZ045
O-744	GTCATGGGGAATCTTTATTTTTTAATGAAAGATCGCCCGGATTAAGTGT AGGCTGGAGCTGCTTC	WZ020
O-745	GTCATGGGGAATCTTTATTTTTTAATGAAAGATCGCCCGGATTATCCG TATGATGTGCCGGATTATGCGTATCCGTATGATGTGCCGGATTATGCG TAAGTGTAGGCTGGAGCTGCTTC	WZ019
